# Antithrombotic effect of repeated doses of the ethanolic extract of local olive (*Olea europaea* L.) leaves in rabbits

**DOI:** 10.3402/ljm.v8i0.20947

**Published:** 2013-05-22

**Authors:** Abdallah M. Dub, Aisha M. Dugani

**Affiliations:** Department of Pharmacology and Clinical Pharmacy, Faculty of Pharmacy, University of Tripoli, Tripoli, Libya

**Keywords:** antithrombotic, thrombosis, OLE, PT, APTT

## Abstract

The incidence of thromboembolic diseases is increasing, and they are a major cause of mortality and morbidity worldwide. Mediterranean diet is known for its high content of olive products, especially olive oil, which has known cardiovascular health benefits, including those on blood pressure, cholesterol level, and thrombogenesis. All previous animal and clinical studies investigating the beneficial antithrombotic effects of olives have focused on olive oil and a few on olive leaves (OLEs). In this study, the ethanolic extract of OLE was evaluated for its antithrombotic activity in the rabbit model of thrombosis induced by ligature of the vena cava and intravenous administration of tissue thromboplastin. Pre-treatment with 100 or 200 mg/kg per day of the ethanolic extract for 8 weeks significantly prolonged the prothrombin time (PT) in comparison to the control group (12.10±0.35 sec and 14.38±0.29 sec vs. 10.8±0.32 sec, *p<*0.05 and 0.001, respectively). In comparison to the control group, the same doses had no statistically significant effect on thrombus weight (16.85±0.67 mg, 16.32±0.35 mg, and 17.81±0.75 mg; *p=*0.18 and 0.06) or on activated partial thromboplastin time (APTT) (19.17±0.33 sec, 19.12±0.73 sec, and 18.97±0.41 sec; *p=*0.36 and 0.43, respectively). One important finding in this study concerns thrombus morphology. In the extract treatment groups, the thrombus was filament-like and did not adhere to blood vessel walls, whereas in the control group the thrombus was thick and almost completely occluded the vein. Therefore, these results suggest that OLE ethanolic extract can modify the extrinsic coagulation pathway as evidenced by the prolongation of PT and changes in thrombus morphology, enough to justify further research to evaluate its possible antithrombotic effects.

Thromboembolic diseases are serious and life threatening. Despite the availability of antithrombotic drugs for the prevention and treatment of arterial and venous thrombosis, thrombotic diseases continue to be a major cause of death and disability worldwide. Therefore, there remains a need for more effective therapies to combat these disorders (1).

In the recent years, there has been a growing interest in using medicinal natural products in the prevention and treatment of many illnesses, including cardiovascular disorders. Olive trees (*Olea europaea* L.) have been widely cultivated in the Mediterranean countries for the production of olive oil. The leaves and fruits contain several constituents with considerable pharmacological activities, including polyphenols, flavones, and flavonoids (2). The most abundant compound in olive leaf (OLE) is oleuropein, followed by hydroxytyrosol (HT), the flavone-seven-glucosides of luteolin and apigenin, and verbascoside (3). Other bioactive constituents of OLE include triterpenes such as oleanolic acid (4) and flavonoids (rutin and diosmin) (5). Extracts of OLEs have been studied both in experimental animals and in humans and have been found to exhibit antimicrobial (6, 7), antidiabetic (8, 9) and anticancer effects (10, 11), and also to possesses beneficial effects on the cardiovascular system, including antihypertensive (12), anti-arrythmic (13), vasodilator (14), hypocholesterolaemic (15), and hypolipidimic activities (16).

A survey of available literature revealed that most of the research work done so far on the antithrombotic effect of the olive plant was on the olive oil. Brzosko et al. (17) were the first to report *in vivo* experimental evidence of the thrombosis prevention properties of olive oil and they suggested that this effect may be mediated by reduced fibrinogen concentrations and impaired platelet–vessel wall interactions. De La Cruz et al. (18) showed that in hypercholesterolaemic rabbits, dietary supplementation with olive oil improved lipid profile and reduced vascular thrombogenicity and platelet activation. Tsantila et al. (19) have reported similar antithrombotic and antitherosclerotic effects of olive oil in rabbits. Larsen et al. (20), in a randomized clinical study on healthy volunteers, have demonstrated that diet rich in olive oil attenuates the acute procoagulant effects of fatty meals.

Therefore, this work was undertaken to investigate the possible haemostatic effects of the ethanolic extract of OLEs using the model of deep vein thrombosis (DVT) and stasis in rabbits.

## Materials and methods

### Plant material and extract preparation

OLEs were collected from the pruning of trees in Ejmail city (north-west Libya) in February 2010. The leaves were cleaned from extraneous matter, shade-dried at room temperature with passive ventilation and crushed into a fine powder. The powder was macerated in ethanol (99%) and allowed to extract for 48 h. The resultant (dark green-brown mixture) was filtered and the filtrate was concentrated in a rotary evaporator under reduced pressure (Stuart). The extract residue was stored in an air-tight, brown glass bottle in a refrigerator. The extraction procedure was carried out in the laboratories of the Libyan National Medical Research Centre (Al-Zawia). The yield of the ethanolic extract was 113.16 g per 1,000 g fresh leaves.

### Animals

Twenty-four healthy, locally inbred adult male white rabbits (2.4–3.1 kg) were used. The animals were maintained on a regular diet purchased from a local company (Tahady Factory, Zawia, Libya) and allowed water *ad libitum*. The rabbits were randomly divided into four groups of six animals each. The study was approved by the Faculty of Pharmacy of Tripoli University, and the experiments were done according to the ethics guidelines of the University.

The drugs and the vehicle (water) were administered by gavage. The first group (positive control) was given warfarin (1.25 mg/kg per day), the second group was given OLE at 100 mg/kg per day, and the third group was given OLE at 200 mg/kg per day. The OLE doses were prepared by reconstitution of the extract residue in distilled water (20 mg/ml). The fourth group served as the negative control and was given distilled water. The treatments were given for 8 weeks.

### Rabbit DVT model

The model of DVT in the rabbit was used and the antithrombotic activity of the extract was evaluated by its effect on weight of the thrombus formed in a section of the vena cava following intravenous injection of thromboplastin combined with stasis. Antithrombotic efficacy was measured as a decrease in the weight of thrombus formed during a 10-min period.

Rabbits were fasted for 12–24 h before the experiments. After shaving and placing the animals in a supine position, they were anesthetized by intramuscular injections of 50 mg/kg of ketamine and 18 mg/kg of xylazine, supplemented as needed using one-half of the full induction dose. Venous thrombosis was induced as previously described (21). The abdomen was opened, the inferior vena cava was exposed by careful separation from the surrounding tissue and isolated between the renal veins and the bifurcation of the inferior vena cava. Two separate pieces of thread were placed under this section of the vena cava, 5.5 cm apart. Thromboplastin (Biolab, France) (0.1 ml/kg) was injected as a bolus in the right femoral vein. Stasis was accomplished first by tying the proximal thread on the vena cava and immediately tying the distal thread. Total time between thromboplastin injection and tying both threads was 18 sec. The stasis was maintained for 10 min and the thrombus was harvested by cutting the vena cava longitudinally. The thrombus was carefully removed, blotted on filter paper, and weighed (22).

### Coagulation analysis and assays

Blood samples were obtained from the marginal ear vein using vacuum blood tubes containing 3.2% trisodium citrate (Vacutest, Arzergrande, Italy) 24 h after the last treatment. Blood was centrifuged at 1,500 rpm for 10 min at room temperature and the plasma was used for determination of prothrombin time (PT) and activated partial thromboplastin time (APTT).

### Prothrombin time

PT was determined using a coagulometer (Coatron M1, Germany). Plasma (25 µl) was pre-warmed for 1 min at 37°C and coagulation was initiated by addition of 50 µl of pre-warmed thromboplastin.

### Activated partial thromboplastin time

APTT was determined using a coagulometer (Coatron M1, Germany). Plasma (25 µl) was incubated for 5 min with 25 µl of APTT reagent, STA-PTT Automate (Diagnostika Stago, Asnieres, France), and coagulation was initiated by addition of 25 µl of pre-warmed calcium chloride solution.

### Statistical analysis

Data were analyzed with MINITAB 14.0. The results are reported as mean values ± SEM. The data were analyzed for statistical differences by a one-way analysis of variance (ANOVA) followed by unpaired Student's *T*-test to determine the level of significance, and *p* values<0.05 were considered significant.

## Results

### Effect of OLE on thrombus weight

Treatment with OLE at 100 or 200 mg/kg per day for 8 weeks had no significant effect (*p>*0.05) on thrombus weight in comparison with the control group treated with distilled water. The values were 16.85 ± 0.67, 16.32 ± 0.35, and 17.81 ± 0.75 mg, respectively. Warfarin (1.25 mg/kg) produced a highly significant (*p<*0.001) decrease in thrombus weight compared with the control group (6.93 ± 0.30 mg and 17.81 ± 0.75 mg, respectively) (Table 1).

**Table 1:** Effect of ethanolic extract of olive leaf (OLE) on thrombus weight in rabbits

Groups	Number of animals	Thrombus weight (mg)
Control (water)	6	17.81 ± 0.75
OLE (100 mg/kg)	6	16.85 ± 0.67
OLE (200 mg/kg)	6	16.32 ± 0.35
Warfarin (1.25 mg/kg)	6	6.93 ± 0.3***

Data are expressed as mean ± SEM (*n=*6),

*** *p<*0.001 compared to control (water) group.

### Effect of OLE on coagulation parameters (PT and APTT)

**Fig. 1:**
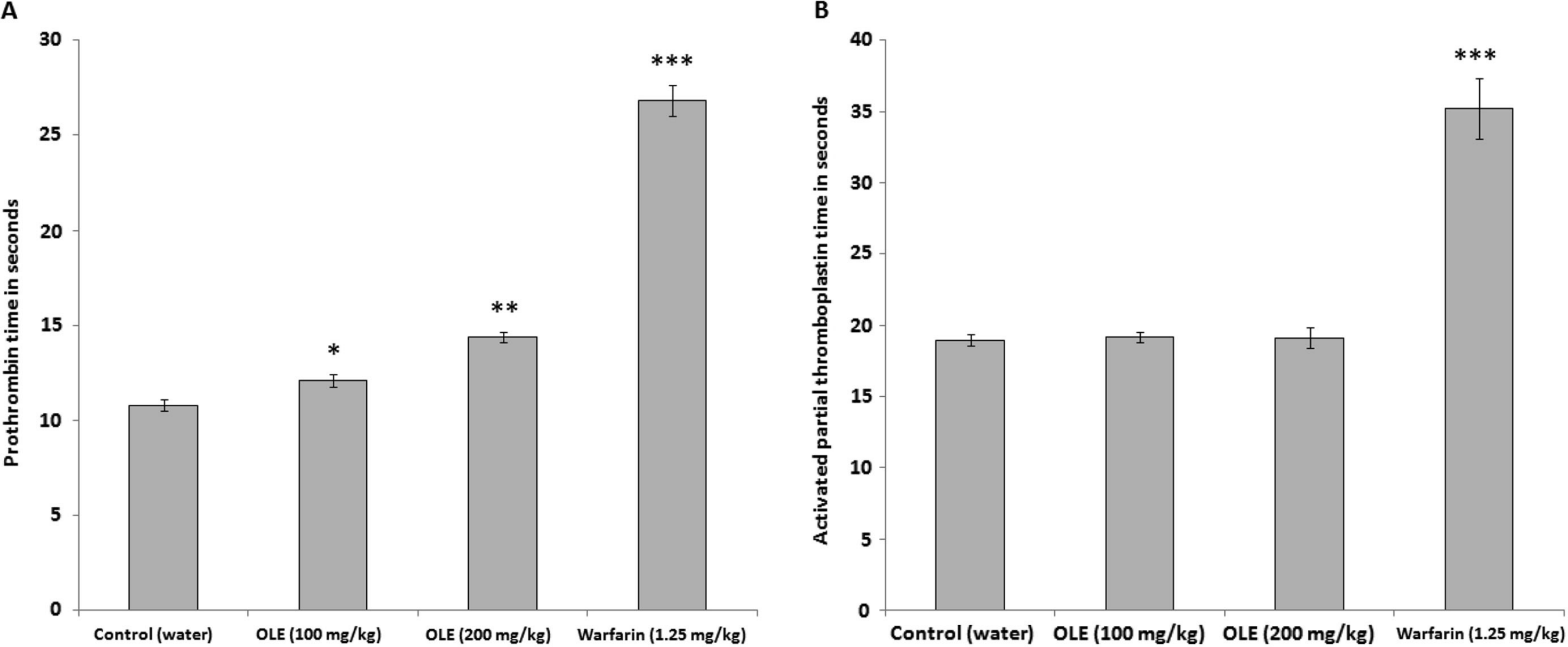
Effect of treatment with ethanolic extract of olive leaf (OLE; 100, 200 mg/kg per day for 8 weeks) on prothrombin time (A) and activated partial thromboplastin time (B) in rabbits. Data are expressed as mean ± SEM (*n=6*), *p<0.05; **p<0.01; ***p<0.001 compared to control (water) group.

To investigate the potential interactions of OLE with coagulation factors, the effects of the extract on coagulation time were evaluated by measuring PT and APTT. In the negative control group, PT was 10.80 ± 0.32 sec. Administration of OLE resulted in a significant prolongation of PT to 12.10 ± 0.35 sec (*p<*0.05) for the dose of 100 mg/kg per day and to 14.38 ± 0.29 sec (*p<*0.01) for the dose of 200 mg/kg per day. Warfarin administration resulted in a significant (*p<*0.001) prolongation of PT to 26.83 ± 0.79 sec as compared to the negative control group given water (Fig. 1A).

The effect of pre-treatment with OLE on APTT is shown in Fig. 1B. Both doses (100 and 200 mg/kg per day) caused only a slight and insignificant increase (*p>*0.05) in APTT when compared to the control (water-treated) group. Warfarin, on the other hand, significantly (*p<*0.001) prolonged APTT as compared to the negative control group (Table 1).

**Fig. 2:**
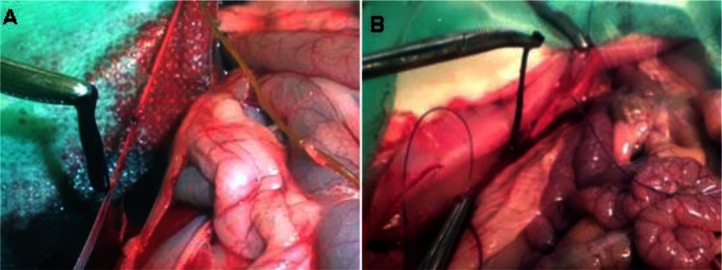
Thrombus harvested from the vena cava of a control rabbit (A) and from a rabbit treated with olive leaf extract (B).

An important observation concerning the morphology of the thrombus is that in the control group treated with water, the thrombus was thick and caused almost a completely blocked vein lumen (Fig. 2A), whereas in the OLE-treated groups the thrombus was thin, filament-like, and elongated (Fig. 2B).

## Discussion

Several antithrombotic agents are available for the control and treatment of thrombotic diseases, but many are expensive and possess toxic effects. It is therefore necessary to search for safe, inexpensive, naturally occurring agents from plants and other natural sources. We show that oral pre-treatment of rabbits with 100 mg or 200 mg/kg OLE for 8 weeks had no effect on APTT (intrinsic coagulation pathway). However, the same doses produced a significant prolongation of PT (extrinsic coagulation pathway). The prolongation of PT was more prominent with the higher dose of 200 mg/kg than the smaller dose (*p<*0.01). This antithrombic activity of OLE might be due to modification of the extrinsic but not the intrinsic coagulation system.

The prolongation of PT only suggests an inhibition of the extrinsic pathway FVII, but mild inhibition of FX, FV, and FII (23). Inhibition of FVII can be explained by a direct effect of nitric oxide (NO) downregulating the factor VII gene (24). Ruano et al. (25) have reported that in hypercholesterolaemic patients, olive oil (rich in phenolic compounds) improved the prothrombotic profile and reduced plasma FVII.

Treatment with OLE caused a slight but statistically insignificant reduction in thrombus weight compared to the control group. The significant prolongation of PT by OLE in our study was not linked to any significant effect on thrombus weight. This result is consistent with the finding of Gitel and Wessler (26), who demonstrated that for a drug to have a significant antithrombotic effect, the PT ratio should be 1.5 or above. Our PT ratios for OLE doses of 100 and 200 mg/kg per day were 1.12 and 1.33, respectively. The only difference was in the shape (morphology) of the thrombus in the different treatment groups. In the negative control group, the thrombus was thick, adherent, and almost occluded the whole vessel, whereas in the treatment groups (both doses), the thrombus was thin and filament-like. This difference in thrombus morphology may be explained by the finding that OLE can cause an increase in the release of NO by the endothelium, preventing platelet adhesion to the vessel wall (27) and also preventing leukocyte adhesion (28). The action of locally produced NO is brief due to its very short half-life and its interaction with haemoglobin, which makes it effective only in the immediate vicinity of its site of production (29). Oleuropein, which is the main constituent of OLE, was found to stimulate nitric acid (NO) production in mouse macrophages and to activate the inducible form of NO synthase (30). Phenolics in OLE probably contribute to the reduction of thrombus adhesion to vessel walls by inhibiting homocysteine-induced endothelial cell adhesion, regardless of their antioxidant activity (31).

Another possible explanation for non-adherence of the thrombi to the vessel walls is that the blood platelets are in motion and in proximity to endothelial cells may become unresponsive to agonists. This inhibition of platelet responsiveness is due to at least three thromboregulatory systems: eicosanoids, endothelial-derived relaxing factor (NO), and an ecto-nucleotidase on endothelial cells that metabolizes the released platelet agonist, ADP. Removal of ADP eliminates platelet recruitment and results in return of platelets to the resting state (32).

In addition, prostacyclin (PGI2) synthesized in the wall of blood vessels could regulate the deposition of platelets on the vascular wall (33). The increase in PGI2 production may be related to the presence of the bioactive molecule, oleanolic acid, in OLE. Oleanolic acid acts as a strong inducer of the synthesis and release of PGI2 in human vascular smooth muscle cells through a cyclooxygenase-dependent mechanism (34).

Other chemical constituents found in OLE, such as flavonoids, polyphenols, and hydrotyrosols, may contribute to its effects on platelet function and thrombogenicity. Hladovec (35) reported that falvonoids are effective ‘protectors’ in two prethrombotic models (venostatic thrombosis and arterial thrombosis) in rats. Polyphenols in OLE have been found to inhibit *in vitro* platelet function in the blood of healthy, non-smoking male volunteers (36). The polyphenols in green tea (37) and *Salvia miltiorrhiza* Bunge (38) exhibit potent antithrombotic effects, probably due to their scavenging of free radicals. Moreover, Léger et al. (39) have shown that HT-rich phenolic extract from olive has an antiaggregating action on platelets and could prevent thrombotic and microthrombotic processes.

There are certain limitations in this study. First, we used crude extract of OLE containing several active constituents. Future studies are needed to investigate the antithrombotic effect of these constituents after their separation. Second, the duration of treatment may be relatively short and another study with longer periods of extract administration may provide different results.

In conclusion, this study reports for the first time that orally administered OLE significantly prolongs PT in the rabbit model of DVT. However, it is premature to draw conclusions about the potential use of OLE as an antithrombotic agent in conditions associated with hyprecoagulopathy state. PT indicates functions of factors II, V, VII, X, and fibrinogen, so it is worthwhile to investigate the effect of OLE on these factors. Future studies will also be needed to determine the effect of OLE on liver function, especially on the synthesis of prothrombin and other procoagulant proteins. Finally, the effect of the extract administration on venous and arterial blood flow may also be evaluated.

## References

[1] Gross PL, Weitz JI (2008). New anticoagulants for treatment of venous thromboembolism. Arterioscler Thromb Vasc Biol.

[2] Gariboldi P, Jommi G, Verotta L (1986). Secoiridoids from *Olea europaea*. Phytochemistry.

[3] Benavente-Garcoa O, Lorente J, Ortuno A, Del Rio JA (2000). Antioxidant activity of phenolics extracted from *Olea europaea* L. leaves Food Chem.

[4] Sato H, Genet C, Strehle A, Thomas C, Lobstein A, Wagner A (2007). Anti-hyperglycemic activity of a TGR5 agonist isolated from *Olea europaea*. Biochem Biophys Res Commun.

[5] Benavente-Garcia O, Castillo J, Lorente J, Alcaraz M (2002). Radioprotective effects *in vivo* of phenolics extracted from *Olea europaea* L. leaves against X-ray-induced chromosomal damage: comparative study versus several flavonoids and sulfur-containing compounds. J Med Food.

[6] Sudjana AN, D'Orazio C, Ryan V, Rasool N, Ng J, Islam N (2009). Antimicrobial activity of commercial *Olea europaea* (olive) leaf extract. Int J Antimicrob Agents.

[7] Markin D, Duek L, Berdicevsky I (2003). In vitro antimicrobial activity of olive leaves. Mycoses.

[8] Gonzalez M, Zarzuelo A, Gamez MJ, Utrilla MP, Jimenez J, Osuna I (1992). Hypoglycemic activity of olive leaf. Planta Med.

[9] Eidi A, Eidi M, Darzi R (2009). Antidiabetic effect of *Olea europaea* L. in normal and diabetic rats. Phytother Res.

[10] Goulas VE, Exarchou V, Troganis V, Psomiadou AN, Fotsis E, Briasoulis E (2009). Phytochemicals in olive-leaf extracts and their antiproliferative activity against cancer and endothelial cells. Mol Nutr Food Res.

[11] Abaza L, Talorete TP, Yamada P, Kurita Y, Zarrouk M, Isoda H (2007). Induction of growth inhibition and differentiation of human leukemia HL-60 cells by a tunisian gerboui olive leaf extract. Biosci Biotechnol Biochem.

[12] Khayyal MT, el-Ghazaly MA, Abdallah DM, Nassar NN, Okpanyi SN, Kreuter MH (2002). Blood pressure lowering effect of an olive leaf extract (Olea europaea) in L-NAME induced hypertension in rats. Arzneimittelforschung.

[13] Dahej ME, Kakoo M, Rezvani ME, Mirhosseini SJ, Moshtaghioon SH (2012). The effects of duration of olive leaf extract administration on ischemia-induced arrhythmias rats. J Mazandaran Univ Med Sci.

[14] Zarzuelo A, Duarte J, Jiménez J, González M, Utrilla MP (1991). Vasodilator effect of olive leaf. Planta Med.

[15] Fki I, Bouaziz M, Sahnoun Z, Sayadi S (2005). Hypocholesterolemic effects of phenolic-rich extracts of Chemlali olive cultivar in rats fed a cholesterol-rich diet. Bioorg Med Chem.

[16] Jemai H, Bouaziz M, Fki I, El Feki A, Sayadi S (2008). Hypolipidimic and antioxidant activities of oleuropein and its hydrolysis derivative-rich extracts from Chemlali olive leaves. Chem Biol Interact.

[17] Brzosko S, De Curtis A, Murzilli S, de Gaetano G, Donati MB, Iacoviello L (2002). Effect of extra virgin olive oil on experimental thrombosis and primary hemostasis in rats. Nutr Metab Cardiovasc Dis.

[18] De La Cruz JP, Villalobos MA, Carmona JA, Martín-Romero M, Smith-Agreda JM, de la Cuesta FS (2000). Antithrombotic potential of olive oil administration in rabbits with elevated cholesterol. Thromb Res.

[19] Tsantila N, Karantonis HC, Perrea DN, Theocharis SE, Iliopoulos DG, Antonopoulou S (2007). Antithrombotic and antiatherosclerotic properties of olive oil and olive pomace polar extracts in rabbits. Mediators Inflamm.

[20] Larsen LF, Jespersen J, Marckmann P (1999). Are olive oil diets antithrombotic? Diets enriched with olive, rapeseed, or sunflower oil affect postprandial factor VII differently. Am J Clin Nutr.

[21] Martin DJ, Toce JA, Anevski PJ, Tollefsen DM, Abendschein DR (1999). Anticoagulant and antithrombotic activity of maltodapoh, a novel sulfated tetrasaccharide. J Pharmacol Exp Ther.

[22] Giardino EC, Costanzo MJ, Kauffman JA, Li QS, Maryanoff BE, Andrade-Gordon P (2000). Antithrombotic properties of rwj-50353, a potent and novel thrombin inhibitor. Thromb Res.

[23] Hood JL, Eby CS (2008). Evaluation of a prolonged prothrombin time. Clin Chem.

[24] De Lucas SBJ, Bartolome J, Carreno V (2003). Nitric oxide downregulates factor VII gene by inhibiting the binding of SP1 and HNF-4. Hepatology.

[25] Ruano J, Lopez-Miranda J, de la Torre R, Delgado-Lista J, Fernandez J, Caballero J (2006). Intake of phenol-rich virgin oil improves the postprandial prothrombic profile in hypercholesterolemic patients. Am J Cli Nutr.

[26] Gitel SN, Wessler S (1983). Dose-dependent antithromboticeffect ofwarfarin in rabbits. Blood.

[27] Tanner FC, Largiader T, Greuert H, Yang Z, Luscher TF (2004). Nitric oxide synthase gene transfer inhibits biological features of bypass graft disease in the human saphenous vein. J Thorac Cardiovasc Surg.

[28] Welch GN, Loscalzo J (1998). Homocysteine and atherothrombosis. N Engl J Med.

[29] Schussheim AE, Fuster V (1997). Thrombosis, antithrombotic agents, and the antithrombotic approach in cardiac disease. Prog Cardiovasc Dis.

[30] Visoli F, Beeosta S, Galli C (1998). Oleuropein, the bitter principle of olives, enhances nitric oxide production by mouse macrophages. Life Sci.

[31] Manna C, Napoli D, Cacciapuoti G, Porcelli M, Zappia V (2009). Olive oil phenolic compounds inhibit homocysteine-induced endothelial cell adhesion regardless of their antioxidant activity. J Agric Food Chem.

[32] Marcus AJ, Broekman JM, Drosopoulos JH, Islam N, Alyonycheva TA, Safier LB (1997). The endothelial cell ecto-ADPase responsible for inhibition of platelet function is CD39. J Clin Invest.

[33] Weiss HJ, Turitto VT (1979). Prostacyclin (prostaglandin I2, PGI2) inhibits platelet adhesion and thrombus formation on subendothelium. Blood.

[34] Martínez-González J, Rodríguez-Rodríguez R, González-Díez M, Rodríguez C, Herrera MD, Ruiz-Gutierrez V (2008). Oleanolic acid induces prostacyclin release in human vascular smooth muscle cells through a cyclooxygenase-2-dependent mechanism. J Nutr.

[35] Hladovec J (1977). Antithrombotic effects of some flavonoids alone and combined with acetylsalicylic acid. Arzneimittelforschung.

[36] Singh I, Mok M, Christensen AM, Turner AH, Hawley JA (2008). The effects of polyphenols in olive leaves on platelet function. Nutr Metab Cardiovasc Dis.

[37] Kang WS, Lim IH, Yuk DY, Chung KH, Park JB, Yoo HS (1999). Antithrombotic activities of green tea catechins and (-)-epigallocatechin gallate. Thromb Res.

[38] Yang SA, Im NK, Ji YJ, Yoo DC, Jhee KH, Lee IS (2008). Radical scavenging and inhibition of platelet function by a polyphenol-rich fraction from *Salvia miltiorrhiza* Bunge. Open Nat Prod J.

[39] Léger CL, Carbonneau MA, Michel F, Mas E, Monnier L, Cristol JP (2005). A thromboxane effect of a hydroxytyrosol-rich olive oil wastewater extract in patients with uncomplicated type I diabetes. Eur J Clin Nutr.

